# Flow cytometric analysis for Ki67 assessment in formalin-fixed paraffin-embedded breast cancer tissue

**DOI:** 10.1186/s12915-024-01980-4

**Published:** 2024-08-26

**Authors:** Natsuki Sato, Masahiko Tsujimoto, Masatoshi Nakatsuji, Hiromi Tsuji, Yuji Sugama, Kenzo Shimazu, Masafumi Shimoda, Hideki Ishihara

**Affiliations:** 1 Nitto Boseki Co., Ltd, 2-4-1, Kojimachi, Chiyoda-ku, Tokyo, 102-8489 Japan; 2 Department of Diagnostic Pathology, Daini Osaka Police Hospital, 2-6-40 Karasugatsuji, Tennoji-Ku, Osaka, 543-8922 Japan; 3 Department of Diagnostic Pathology, Osaka Police Hospital, 10-31 Kitayamacho, Tennoji-Ku, Osaka, Japan; 4 Department of Breast and Endocrine Surgery, Osaka University Graduate School of Medicine, 2-2 Yamadaoka, Suita, Osaka, 565-0871 Japan; 5 Present Address: Osaka Pathology and Cytology Laboratory, 2-2-26 Kunijima, Higashiyodogawa-Ku, Osaka, 533-0024 Japan; 6 Department of Pathobiochemistry, Faculty of Pharmacy, Osaka Medical and Pharmaceutical University, 4-20-1 Nasahara, Takatsuki, Osaka, 569-1094 Japan; 7 Department of Research Support, National Institutes of Biomedical Innovation, Health and Nutrition, 7-6-8, Saito-Asagi, Ibaraki City, Osaka, 567-0085 Japan

**Keywords:** Ki67 labelling index, Immunohistochemistry, Breast cancer, Formalin-fixed paraffin-embedded tissue, Flow cytometry

## Abstract

**Background:**

Pathologists commonly employ the Ki67 immunohistochemistry labelling index (LI) when deciding appropriate therapeutic strategies for patients with breast cancer. However, despite several attempts at standardizing the Ki67 LI, inter-observer and inter-laboratory bias remain problematic. We developed a flow cytometric assay that employed tissue dissociation, enzymatic treatment and a gating process to analyse Ki67 in formalin-fixed paraffin-embedded (FFPE) breast cancer tissue.

**Results:**

We demonstrated that mechanical homogenizations combined with thrombin treatment can be used to recover efficiently intact single-cell nuclei from FFPE breast cancer tissue. Ki67 in the recovered cell nuclei retained reactivity against the MIB-1 antibody, which has been widely used in clinical settings. Additionally, since the method did not alter the nucleoskeletal structure of tissues, the nuclei of cancer cells can be enriched in data analysis based on differences in size and complexity of nuclei of lymphocytes and normal mammary cells. In a clinical study using the developed protocol, Ki67 positivity was correlated with the Ki67 LI obtained by hot spot analysis by a pathologist in Japan (rho = 0.756, *P* < 0.0001). The number of cancer cell nuclei subjected to the analysis in our assay was more than twice the number routinely checked by pathologists in clinical settings.

**Conclusions:**

The findings of this study showed the application of this new flow cytometry method could potentially be used to standardize Ki67 assessments in breast cancer.

**Supplementary Information:**

The online version contains supplementary material available at 10.1186/s12915-024-01980-4.

## Background

The nuclear protein Ki67 is expressed in cells at all stages of the cell cycle except for G_0_ phase [1, 2]. The protein functions as a biological surfactant that disperses chromosomes during cell division [3] and it is also involved in ribosomal RNA transcription [4]. Ki67 has been used extensively as a biomarker for estimating cell proliferation [5]. The Ki67 labelling index (LI), which reflects the proportion of Ki67-expressing cells in cancer cells, is used routinely for determination of adjuvant chemotherapy in hormone receptor-positive breast cancer [5, 6]. In 2021, a Ki67 immunohistochemistry (IHC) assay with an LI cut-off of 20% was approved by the Food and Drug Administration as a companion diagnostic for combination with abemaciclib, a cyclin-dependent kinase 4/6 inhibitor, and endocrine therapy in hormone receptor-positive, human epidermal growth factor receptor 2 (HER2)-negative, node-positive, early breast cancer [7]. Estimating Ki67 LI values is typically based on unaided microscopic examinations using an IHC-stained slide with the anti-Ki67 antibody represented by clone MIB-1. However, inter-observer and inter-laboratory variations have been reported in these visual assessments [8]. Indeed, a variety of factors have been shown to affect the interpretation of these assessments, including the conditions under which the cancer tissue was fixed and the protocols employed from pretreatment to staining [9], pathologist assessments and their selection of tumour regions [10], counting methods [11] and the cut-off intensity of stained cells. Although differences in staining properties due to antibody clones have been reported [12, 13], the most widely used antibody appears to be MIB-1, which was established by Gerdes et al. at the University of Kiel [14]. To address these issues, The International Ki67 in Breast Cancer Working Group of the Breast International Group, and the North American Breast Cancer Group (BIG-NABCG) conducted studies on standardizing assessment methods for use in visual analysis. As a result of these efforts, the intraclass correlation coefficient (ICC) among observers was improved from 0.59 (95% confidence interval (CI) = 0.47–0.78) to 0.87 (95% CI = 0.799–0.93) [15–18]. The adoption of centralized staining and training methods, including calibration to avoid inter-observer and inter-laboratory variation, was demanded though these modifications enabled more robust scoring. In addition, recent improvements in imaging technology and the use of ultra-high definition displays have facilitated considerable advances in observations and analysis in digital pathology [19–21] with some reports proposing that artificial intelligence (AI) could be applied to microscope-based analysis of Ki67 [22]. However, due to differences among scanners, software and operators, the application of new commercially available platforms with or without AI could cause new variations in the interpretation of data. To date, although good ICC among three open-source software programs for digital pathology image analysis has been observed (ICC = 0.933, 95% CI = 0.879–0.966) [23], the spread of platforms and the quality and extent of machine-learning in the underlying AI are crucial factors that may affect the consistency of assessments. In addition, fundamental differences among laboratories in the methods used to stain slides have still not been resolved.

Flow cytometry (FCM) is a widely used cell biology technique, and immunophenotyping by FCM has become a standard procedure for evaluating and monitoring hematopoietic tumours in clinical settings [24, 25]. The application of solid tumours to FCM is not as widespread as hematopoietic tumours, primarily due to the requirement for tissue dissociation. Even if single cells have been well recovered from tissue specimens, using biomarkers to screen and sort cancer cells should be employed because of contamination by cells other than cancer cells, such as stromal cells and lymphocytes. In addition, when formalin-fixed paraffin-embedded (FFPE) tissue is used as a sample, the antigen retrieval method of formalin-crosslinked antigens is essential. The modified conditions should be verified instead of simply relying on previous conditions for parameters such as temperature, reaction time and pH [26], as single cells subjected to FCM differ from those in sections used for IHC. Leers et al. described flowcytometric detection of Ki67 using four antibody clones with an FFPE breast cancer tissue [27]. Their findings showed that three of the antibodies tested, including MIB-1, did not react with Ki67. Establishment of an FCM protocol to detect Ki67 in FFPE breast cancer tissue is expected to contribute to the standardization of Ki67 assay.

In this study, we developed an observer-independent method for the assessment of Ki67 using FCM. MIB-1 antibody, which has been widely used in clinical settings, was used for the experiments. A combination of mechanical dissociations and thrombin treatment enabled single-cell nuclei to be recovered efficiently from FFPE breast cancer tissue. Unlike enzymatic dissociation, the integrity of the nucleoskeleton of cell nuclei was retained, which facilitated the enrichment of cancer cell nuclei on forward scatter (FSC)-side scatter (SSC) scatterplots. Ki67 positivity obtained using our method was well correlated with pathological Ki67 LI.

## Results

### Optimal selection of enzymes to dissociate tissue is essential for flow cytometric analysis of Ki67 in an FFPE breast *cancer* tissue with MIB-1 antibody

**Fig. 1:**
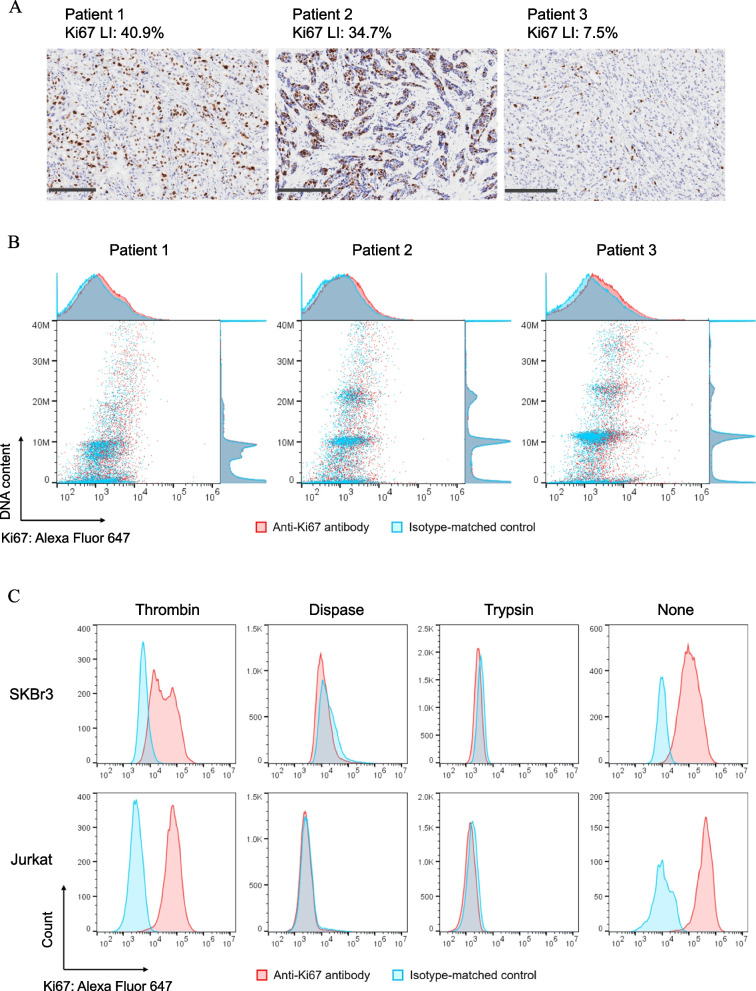
Flowcytometric detection of Ki67 in cells recovered from FFPE breast cancer tissues by enzymatic dissociation. **A** Ki67 expression in breast cancer by IHC. Brown nuclear stain showing Ki67-positive tumour cells. Scale bars are 200 µm. **B** Bivariate flowcytometric analysis of Ki67 (*X*-axis) and DNA content (*X*-axis). Cells were recovered from FFPE tissues treated by 0.25 ml of dispase reagent (25 mM Tris–HCl pH 7.4, 150 mM NaCl, 3000 PU/l dispase (FUJIFILM Wako Pure Chemical Corporation, Osaka, Japan)) at 37 °C for 20 min after different pretreatments. No gating was performed in the data analysis. Red dots and curves show cells incubated with MIB-1 antibody. Blue dots and curves correspond to the background level of cells treated with isotype-matched control. **C** FCM of Ki67 in SKBr3 cell lines treated without (right) or with thrombin reagent (left), dispase reagent (middle left) and trypsin reagent (25 mM Tris–HCl pH 7.4, 150 mM NaCl, 2.5 mg/ml trypsin (Worthington Biochemical Corporation, Lakewood, NJ, USA)) (middle right). Gating scheme to eliminate debris is shown in Additional file 1: Fig. S3A. Red curves show cells incubated with MIB-1 antibody. Blue curves correspond to the background level of cells treated with isotype-matched control

Since digestive enzymes can degrade the proteins responsible for the adhesion of cells within the extracellular matrix [28], they have been widely used for tissue dissociation. We therefore evaluated the effect of dispase and trypsin on the dissociation of FFPE tissue. Three FFPE breast cancer tissue samples with different Ki67 LI levels were analysed by FCM analysis after different pretreatment methods, including enzymatic dissociation (Fig. 1A, B and Additional file 1: Fig. S1). However, Ki67 could not be detected regardless of the original LIs (7.5–40.9%). Dispase cleaves the N-terminal region of neutral and nonpolar amino acids [28, 29], and trypsin cleaves peptide bonds of the C-terminal regions of lysine and arginine residues [30]. The number of cleavages by dispase and trypsin in the whole Ki67 sequence was 2183 and 530 in isoform 1, and 1931 and 480 in isoform 2, respectively (Additional file 1: Fig. S2A) [31]. These enzymes also cleaved the epitope of the MIB-1 antibody (TPKEKAQALEDLAGFKELFQT) [32]. We considered that the following three factors were essential for selecting an enzyme for tissue dissociation in FCM analysis of Ki67 using MIB-1 antibody: (1) no cleavage of the MIB-1 epitope; (2) cleavage of the CK cytoskeleton to increase antibody accessibility; (3) cleavage of collagen to isolate cells. We conducted in silico experiments to estimate the number of cleavages that typically occur in cytokeratin (CK5, 7, 8, 14, 17, 18 and 19) [33–35] and collagen (collagen type I α1 chain, α2 chain and type III α1 chain) [36] subtypes expressed in normal mammary gland and breast cancer tissue by representative digestive enzymes (Additional file 1: Fig. S2B and S2C). Enzymes that matched these three requirements were clostripain, proline endopeptidase and thrombin. We did not select clostripain because it was reported to cleave the C-terminal region of lysine to a minor degree [37]. To confirm whether Ki67 can be detected by MIB-1 antibody in cells treated with thrombin and proline endopeptidase, we next used formalin-fixed cell lines (Fig. 1C and Additional file 1: Fig. S3B). Ki67 could be detected both without enzyme treatment and after treatment with the selected enzymes. Dispase and trypsin reproduced the FFPE tissue results of undetectable of Ki67, suggesting that dissociation of the FFPE tissue critical for detecting Ki67 by flow cytometry using the MIB-1 antibody. In addition, to extend the application to other nuclear proteins, we investigated the detection of oestrogen receptors (ER) and progesterone receptors (PgR), which are necessary for breast cancer. The clones EP1 [38] and PgR1294 [39] were used to detect ER and PgR, respectively, since they are clinically available antibodies. As shown in Additional file Fig. S4, ER and PgR were specifically detected.

### Combination of rotary and ultrasonic homogenization improved the recovery of single-cell nuclei from an FFPE tissue

**Fig. 2:**
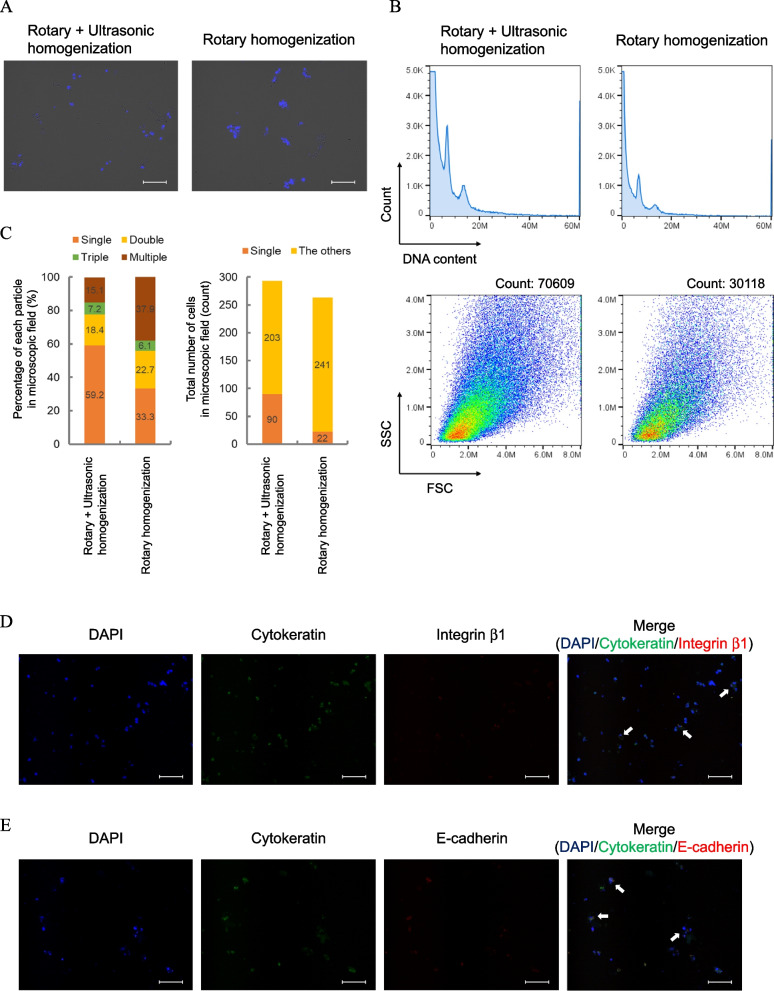
Recovery of intact cell nuclei from FFPE breast cancer tissues after mechanical dissociation.** A** Bright-field and fluorescence merged microscope images of DAPI-stained particles with a combination of rotary and ultrasonic homogenization (left) and rotary homogenization only (right). Scale bars are 100 µm. Images show representative cells. **B** Changes in the number of particles and distribution according to combination of rotary and ultrasonic homogenization (left) and rotary homogenization only (right). DNA content chart without gating (top) and the FSC-SSC scatterplots after gating of ≥ 2n (bottom) are shown. **C** Percentage of single cells and particles with aggregated of 2, 3 or more than 3 cells (left), and the number of single cells in the total number of cells (right). The cells (or particles) were counted in eight separate fields of the microscope view. ImageJ was used for counting. **D**,** E** Fluorescence microscopy images of cells recovered from FFPE tissues after mechanical dissociation. Cells were stained for nuclei (DAPI; blue), cytokeratin (Alexa Fluor 488; green), integrin β1 (**D**, Alexa Fluor 647; red) and E-cadherin (**E**, Alexa Fluor 647; red). Scale bars are 100 µm. Images show representative cells. White arrows show aggregates of tissue debris

Although the selected enzymes did not interfere with the detection of Ki67 by the MIB-1 antibody, the enzymes alone were unable to dissociate FFPE tissues. We therefore investigated the addition of mechanical dissociation to better dissociate the tissues. To optimise conditions for this process, the following three parameters were examined based on particle counts and the range of DNA contents; cell recovery rate: 2n/total count × 100, debris rate: < 2n/2n and single cell rate: 2n/ ≥ 2n × 100 (Additional file 1: Fig. S5A). Regarding the optimization of rotation speed, rotary homogenizer (RP-10 approved as a medical device in Japan) showed the best performance in terms of the total count of recovered particles, cell recovery rate and debris rate (Additional file 1: Fig. S5B). However, since the disruption of many tissues was insufficient with the RP-10 rotary homogenizer alone (Fig. 2A right), an ultrasonic homogenizer was also used. Although the total count of recovered particles increased in proportion to the amplitude of ultrasonic waves, the cell recovery rate decreased and the debris rate increased (Additional file 1: Fig. S5C). A larger variation in the total count of recovered particles was observed at an amplitude of 30% compared to an amplitude of 20% (Additional file 1: Fig. S5D and S5E). The single cell rate was constant after 1 min. Additional experiments showed that a prolonged treatment time by sonication led to the destruction of cells and caused the aggregation of cells due to exposed DNA (Additional file 1: Fig. S6A and S6B). Thus, the combination of rotary and ultrasonic homogenizers recovered more than twice of the number of cells compared to the rotary homogenizer alone (Fig. 2A), and the recovery of single cells was also improved (Fig. 2B and C). Ultrasonic homogenization by itself almost never dissociated FFPE tissue (Additional file 1: Fig. S7).

To observe the state of recovered cells, represent cell membrane proteins, such as integrin β1 (Fig. 2D) and E-cadherin (Fig. 2E), and CK as a cytoskeleton protein were stained. CK in recovered single cells was stained; however, membrane proteins were not. Interestingly, membrane protein staining was observed in aggregates of tissue debris. Similar results were obtained for the HER2 protein (Additional file 1: Fig. S8), which is essential for determining treatment strategies for breast cancer patients. These results suggested that our combined mechanical dissociation method could recover cell nuclei without cell membrane.

### Effect of thrombin treatment on enhancing the stainability of Ki67

**Fig. 3:**
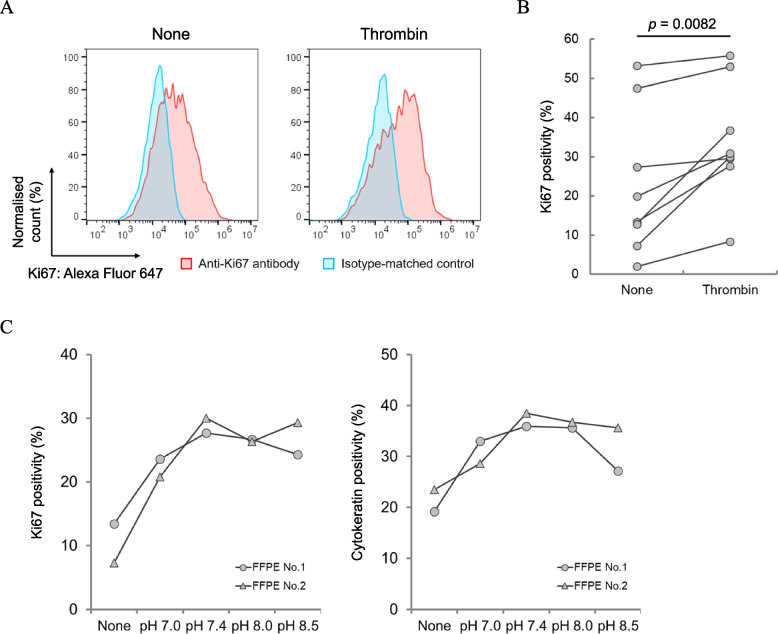
Improvement of Ki67 stainability by thrombin treatment. **A** Representative charts of Ki67-stained cell nuclei obtained from FFPE breast cancer tissues without or with thrombin treatment. Red curves showed cell nuclei incubated with MIB-1 antibody. Blue curves correspond to the background level of cell nuclei reacted with an isotype-matched control. **B** Change in the positivity of Ki67 with or without thrombin treatment. Two serial sections from eight cases were used for this experiment. Positivity was calculated as the proportion of Ki67-positive cell nuclei in total cell nuclei. The 95th percentile of isotype-matched control was used for the judgement of Ki67-negative and Ki67-positive cell nuclei. *P*-values for significant differences between groups were calculated using a paired student *t*-test. **C** pH dependency of thrombin treatment. The hydrophilized FFPE tissue was incubated with thrombin in TBS buffer under different pH conditions at 37 °C for 20 min. Cell nuclei from FFPE tissues were double-stained with MIB-1 and pan-cytokeratin antibodies

After establishing a method for tissue dissociation, we attempted to detect Ki67 using MIB-1 antibody by FCM. We also treated hydrated sections with thrombin which did not affect the detection of Ki67 (Fig. 1C). As shown in Fig. 3A, Ki67 was detected, and interestingly, a shift in the peak of MIB-1 antibody staining was observed by the addition of thrombin treatment. Ki67 positivity, which was calculated using the 95th percentile of the isotype-matched control as a cut-off, was significantly increased in the thrombin-treated group (Fig. 3B). To examine the pH dependency of the effect of thrombin on the stainability, serial sectioned-FFPE tissues were incubated in thrombin reagent adjusted to pHs ranging from 7.0 to 8.5. The Ki67 positivity peaked at pH 7.4 (Fig. 3C, left). This enhancement was also observed with cytokeratin staining (Fig. 3C, right). However, the effect of treatment with proline endopeptidase on the stainability was markedly less than that observed with thrombin (Additional file 1: Fig. S9). These data suggest that the enzymatic ability of thrombin affected the stainability of Ki67.

### The Ki67 positivity in cytokeratin-positive populations showed a strong correlation with Ki67 LI

**Fig. 4:**
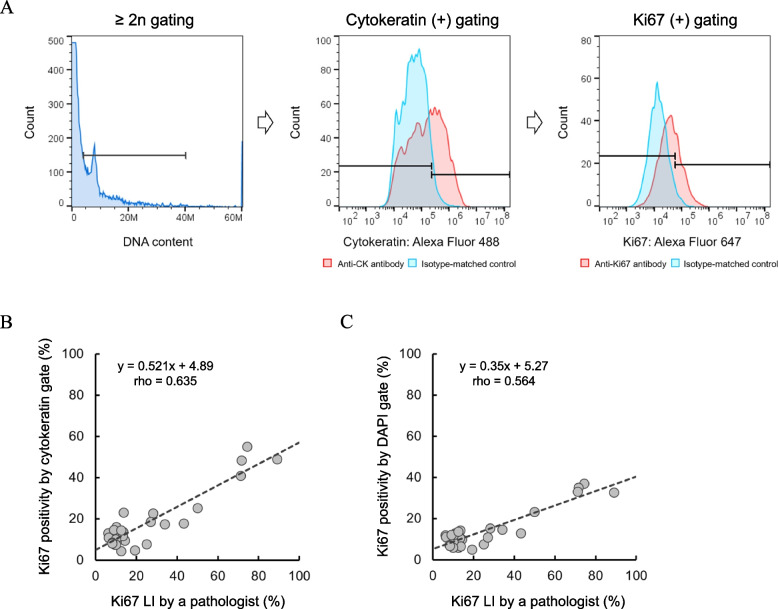
Comparison of percentage of Ki67 positive cell nuclei among cytokeratin positive cell nuclei by FCM and the Ki67 LI. **A** Gating scheme for the calculation of the Ki67 positivity in cytokeratin-positive cell nuclei. The population was initially gated in the region with a 2n peak and more than 2n on a DNA content chart, and debris was eliminated. Then, cytokeratin-positive cell nuclei were defined as a cut-off at the 95.^th^ percentile of an isotype-matched control. **B**,** C** Correlation (Spearman’s) between the Ki67 LI and Ki67 positivity by CK gating (**B**) or DAPI 2n gating only (**C**) in FFPE breast cancer tissues (*n* = 30)

The Ki67 LI basically counts Ki67-positive cells in cancer cells of the invasive region [5]. CK is expressed in approximately 90% of breast cancers regardless of the subtype and is used for functional classification and prognostic indication [33, 40, 41]. As shown in Fig. 4A, we employed three gating steps using CK to estimate Ki67 positivity in cancer cells. The first gate was performed to screen for cell nuclei and eliminate debris, the second CK gate was used to enrich cancer cell nuclei. Ki67 positivity, which was calculated based on the last gate, correlated strongly with the Ki67 LI (*y* = 0.521x + 4.89, rho = 0.635, *P* = 0.0002) (Fig. 4B). Assessments of Ki67 positivity without CK gating also showed a good correlation with the Ki67 LI; however, the slope was low compared to that obtained using the CK gate (*y* = 0.350x + 5.27, rho = 0.564, *P* = 0.0012) (Fig. 4C).

### Gating specific regions of FSC-SSC scatterplots improved the correlation with Ki67 LI

**Fig. 5:**
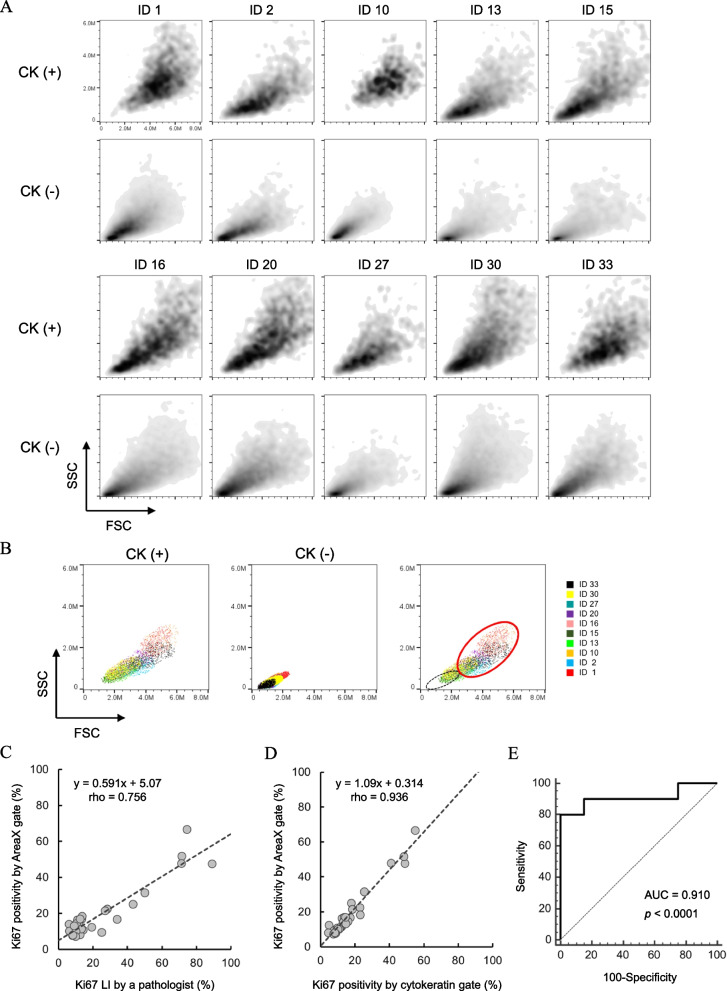
Verification of Ki67 positivity without cytokeratin gating.** A** Distribution of CK-positive and -negative cell nuclei on the FSC-SSC scatterplots in representative cases (*n* = 10).** B** Overlay of the main population of CK-positive and CK-negative cell nuclei on the FSC-SSC scatterplots. The main population was defined as the region where about 40% of the cell nuclei were concentrated in the entire area. The red oval was set as the gate to exclude CK-negative cell nuclei shown by the black dashed oval. **C** Correlation (Spearman’s) between the Ki67 LI and the Ki67 positivity by AreaX gating. **D** Correlation (Spearman’s) of Ki67 positivity between AreaX and CK gating. **E** ROC curve analysis of the FCM method for diagnosis of Ki67. Twenty per cent of the Ki67 LI was utilized as the cut-off of Ki67 positive and negative

**Fig. 6:**
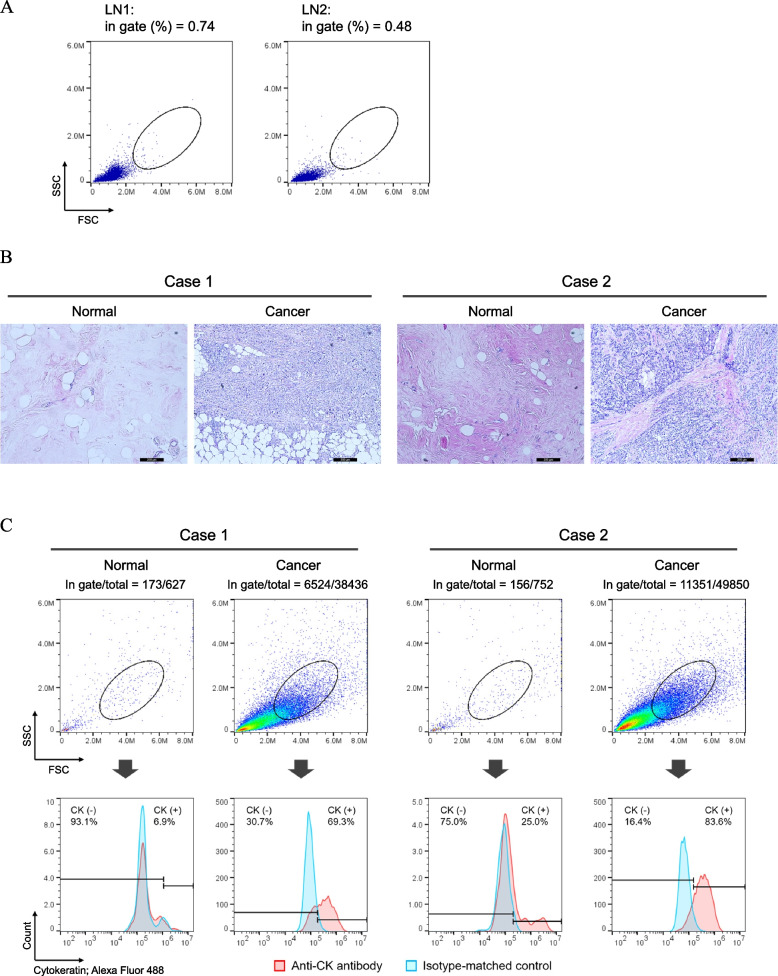
Contamination of cell nuclei derived from normal lymph node or normal epithelial tissue to AreaX. **A** Lymphocyte nuclei obtained from normal lymph node FFPE sections were stained with DAPI. The main population was used to produce the FSC-SSC scatterplots after removal of the debris on the DAPI content chart. The percentage of cell nuclei included within the gate was calculated using FlowJo software. **B** Representative H&E-stained normal and cancer breast sections. Scale bars are 200 µm. **C** Distribution of cell nuclei from normal and cancer breast tissues from the same patient, and CK status within AreaX. Population was initially gated in the region with a 2n peak and more than 2n on the DNA content chart and then indicated on the FSC-SSC scatterplots (top). CK status of cell nuclei in AreaX was verified by overlaying the charts obtained for the CK antibody and its isotype-matched control (bottom)

Though the gating method using CK showed a good correlation with the Ki67 LI, contamination of normal epithelial cells is controversial. In addition, low or no CK-expressed cells induced by epithelial-mesenchymal transition have been reported in breast cancer tissues [42], and breast cancer patients that are clinically CK-negative are also known to occur at a certain prevalence rate [43, 44]. To investigate whether cancer cells can be enriched without using biomarkers, we first checked the distribution of CK ( +) and CK ( −) cells on the FSC-SSC scatterplots (Fig. 5A). The main populations in which cell nuclei were concentrated could be separated into those that were CK ( +) and those that were CK ( −). Our method facilitated the recovery of cell nuclei by mechanical dissociation of the tissue and did not chemically disrupt the peptide bonds, thus preserving the lamin proteins associated with the nucleoskeletal structure [45] (Additional file 1: Fig. S10A). Most cells removed by CK gating were tumour-infiltrated lymphocytes (TIL) [46]. The retention of the nucleoskeleton did not cause a change in the size of cell nuclei, which facilitated the separation of lymphocytes on the FSC-SSC scatterplots (Additional file 1: Fig. S10B and S10C). We therefore created a new gate named AreaX to avoid CK ( −) and mostly include CK ( +) on the FSC-SSC scatterplots (Fig. 5B). The scheme incorporating the AreaX gate instead of the CK gate to calculate Ki67 positivity is shown in Additional file 1: Fig. S11. AreaX-gating improved the correlation coefficient with the Ki67 LI (*y* = 0.591x + 5.07, rho = 0.756, *P* < 0.0001) (Fig. 5C), and showed 9% higher positivity compared to CK gating (y = 1.09x + 0.314, rho = 0.936, *P* < 0.0001) (Fig. 5D). Using 20% of the Ki67 LI as a positive and negative cut-off, our method showed a statistically significant ROC-AUC (AUC = 0.910, *P* < 0.0001, Youden index = 18.5). Our assay, which include pretreatment of FFPE tissue and analysis of FCM, showed good reproducibility assessed around the Youden index as CV = 9.2% (mean = 19.2) (Additional file 2: Table S1), and the number of cell nuclei in AreaX for the evaluation was comparable to cytokeratin gating (Additional file 2: Table S2). We therefore analysed the extent contamination by lymphocytes and normal epithelium to refer to the method for increasing the number of cancer cell nuclei in AreaX. The rates of contamination by lymphocytes in AreaX were 0.74% in LN1 and 0.48% in LN2, respectively (Fig. 6A). Interference of normal epithelial cells was verified by using normal breast tissues and cancer tissues resected from the same patient (Fig. 6B and Additional file 1: Fig. S12). The findings showed that the number of cell nuclei from normal and cancer tissues contained in AreaX was 141 and 10,506 in Case 1, and 144 and 11,618 in Case 2, respectively, for a cross-sectional area of 100 mm^2^ (Fig. 6C). In addition, most of the normal tissue-derived cell nuclei in AreaX were CK-negative.

## Discussion

In this study, we demonstrated the efficacy of combining mechanical homogenization methods with thrombin treatment to efficiently recover single-cell nuclei for flow cytometry analysis of Ki67 in breast cancer FFPE tissue. The Ki67 in the recovered cell nuclei retained reactivity against the MIB-1 antibody. Similarly, since the nucleoskeletal structure was preserved, it was possible to enrich cancer cell nuclei by FSC (particle size) and SSC (particle complexity) in the analysis. The findings of the developed assay were strongly correlated with those based on Ki67 LI performed by a pathologist.

We conducted extensive tissue dissociation assessments of different sample pretreatment methods for flow cytometric analysis of Ki67 (Fig. 2, Additional file 1: Fig. S5–7). Enzymatic dissociation, such as that with trypsin, dispase, collagenase or their combinations [47, 48], has been widely used in tissue-based experimental protocols due to the simplicity and uniformity of sample processing [28]. Thrombin and proline endopeptidases did not cleave the epitope of MIB-1 and had cleavage sites in collagens (Additional file 1: Fig. S2C). No Ki67 was detectable in cell lines exposed to type IV collagenase treatment (Additional file 1: Fig. S13), indicating that efficient tissue dissociation cannot be achieved with enzymes alone. We therefore replaced the treatment steps using trypsin or dispase in combination with mechanical homogenizers. In addition, ultrasonic homogenization was added to our established mechanical dissociation conditions to recover single cells more efficiently (Fig. 2A–C). Although the optimal condition at a sonication step was relatively narrow, in which the cells were destroyed when the duration of sonication was too long (Additional file 1: Fig. S6), we successfully detected Ki67 in FFPE breast cancer tissue.

Surprisingly, thrombin treatment enhanced Ki67 stainability (Fig. 3A and B). The number of thrombin cleavage sites in Ki67 was three in isoform 1 and two in isoform 2 (Additional file 1: Fig. S2A). A few nicks in Ki67 might result in increased efficiency of antigen retrieval and some cleavage in CK may lead to improvement of antibody accessibility. However, in addition to confirming whether a similar effect is observed with proline endopeptidase, such modifications will need to be verified in future (Additional file 1: Fig. S9).

Although Ki67 assessment methods have not yet been standardized, studies conducted mainly by BIG-NABCG have shown promising results with the educational application tools and counting methods employed in IHC analysis [6]. In recent years, the field of digital pathology has developed rapidly with the spread of AI [49]. Our assay, which employs optical measurements by FCM is robust, and anyone can perform all of the protocol steps, including pretreatment. The Ki67 positivity estimates obtained using our method showed a good correlation with Ki67 LI (rho = 0.635), although the obtained values were approximately 40% lower than those obtained by hotspot analysis in Japan (Fig. 5C). The reason why our results were lower than those obtained by a pathological Ki67 LI is because we measured Ki67-positive cancer cells in the entire section against the hot spot analysis of a pathologist. Previous studies have shown that the bias between average and hotspot methods in IHC studies was approximately 30% in luminal breast cancer [50, 51]. Our results were also comparable to these previous studies, and a comparative study with multiple pathologists should be conducted in the future.

We also describe a gating method for estimating Ki67 positivity. Three gating steps were employed in this study (Additional file 1: Fig. S11). Intact cell nuclei were selected by gating above the peak corresponding to 2n in the DNA content chart. Next, using the retained nucleoskeletons, we selected regions where the number of cell nuclei was concentrated while excluding TILs based on FSC (particle size) and SSC (particle complexity) (Fig. 6A). Enrichment of cancer cells using the FSC-SSC scatterplots (AreaX) could be used to recover cells with low or no CK-expressed cells that would be eliminated by the CK gate. This AreaX gate caused a marked 9% increase in the positivity rate of Ki67 by compared to the CK gate (Fig. 5D). Although vascular endothelial cells, fibroblasts and adipocytes were observed in H&E-stained images (Fig. 6B), few cells were recovered from normal mammary glands using our method, and almost no contamination in AreaX was observed (Fig. 6C). Pathologists typically identify cancer cells based on nuclear atypia, such as characteristics based on size, morphology and chromatin homogeneity in a staining image [52]. Since we also perform gating using the FSC-SSC scatterplots to distinguish the morphology of cancer cell nuclei, this approach is fundamentally equivalent to the visual observation conducted by pathologists. The prospect of biomarker-free enrichment of cancer cell nuclei could be to rescue samples from patients with low proportions of nucleated tumour cells, even after laser macrodissection, and who are not well suited for a genomic profiling assay [53].

The average number of cell nuclei yielded using our method with three 20-µm sections was 1,885 (Additional file 2: Table S2) (see the “Methods” section) from entire tumour tissues without selection bias of observation area, which is twice the number of cells checked by pathologists (500 to 1000 invasive cancer cells) [11]. These suggest that our method could be more accurate for estimating Ki67 positivity than estimates by pathologists.

Molecular subtyping of breast cancer patients based on ER, PgR, HER2 and Ki67 status is of great importance and is routinely used for the stratification of treatment personalization and prognostication [54]. Since our pretreatment method could only efficiently recover cell nuclei without cell membrane (Fig. 2D, E and Additional file 1: Fig. S8), the detection of three biomarkers except HER2 was achieved (Additional file Fig. S4). ER and PgR will be necessary to compare tissue specimens evaluated by pathologists with our assays in the future.

## Conclusions

Our findings in this study suggest that mechanical tissue dissociation combined with thrombin treatment, which has little effect on the chemical structure of the target protein, expands antibody options, retains nucleoskeletal integrity and enriches cancer cells without the need for a biomarker. Although there is a limitation in a clinical study with one pathologist using a limited number of cases, our study successfully showed a flow cytometry method with the newly developed pretreatment process of FFPE specimens could potentially be used to standardize Ki67 assessments in breast cancer. The methodology should be in the near future assessed in a larger, multi-centre study compared to standard pathologic assessment.

## Methods

### Cell culture and fixation

MDA-MB-231, T47D and SKBr3 human breast cancer cell lines and Jurkat human T-cell lymphoma cell lines were purchased from ATCC (Manassas, VA, USA). Cells were cultured according to the suppliers’ instructions. MDA-MB-231 was incubated in a humidified atmosphere without CO_2_ at 37 °C, and the other lines were incubated under 5% CO_2_. Cells were cultured until fully confluent, washed with phosphate-buffered saline (PBS), and then harvested. After suspending the cells in PBS, 1 ml aliquots of 1 × 10^6^ cells were centrifuged at 200 × *g* followed by resuspension of the pellets in 10% neutral buffered formalin solution (FUJIFILM Wako Pure Chemical Corp., Osaka, Japan), and the samples were incubated at 4 °C for 24 h. Prior to use in the experiments, the formalin solution was removed and the cells were washed with PBS.

### Tissue specimens

FFPE tissues from patients (*n* = 30) with breast carcinoma who underwent surgery at Osaka Police Hospital in 2010 were used in this study. Tumour characteristics were assessed by haematoxylin and eosin (H&E) staining. Clinicopathological data of patients are shown in Additional file 2: Table S3. The tissues were compared by IHC and FCM. This study was approved by the institutional review board of Osaka Police Hospital (Approval No.: 947) and Nitto Boseki Co., Ltd. (Approval No.: EC-158058). FFPE tissues purchased from ProteoGenex, Inc. (Inglewood, CA, USA) were used for optimizing study conditions.

### Immunohistochemistry and estimation of Ki67

Three micrometres slides were cut from paraffin blocks, which contained formalin-fixed breast tumour tissue. During the whole staining procedure the slides were treated with the fully automated Ventana BenchMark ULTRA staining system (Roche Holding AG, Basel, Switzerland). Ki67 antigen retrieval was accomplished by pretreatment on the Ventana BenchMark ULTRA using CC1 buffer. The primary antibody was Ki67 (clone MIB-1, 1:50 dilution; Agilent Technologies, Inc., Santa Clara, CA, USA). The ultraView DAB universal kit (Roche Holding AG) was used for detection. The slides were counterstained with hematoxylin and a bluing reagent. The stained slides were scanned on the Hamamatsu whole-slide scanner (NanoZoomer-XR, Hamamatsu Photonics K.K., Shizuoka, Japan) and analyzed using the software package NDP.anlyze (Hamamatsu Photonics K.K.). The observation site was the hot spot of the invasive lesion, and regions containing at least 1000 cancer cells were evaluated. The NDP analysis was set as follows: (1) the setting for “Detection of Nuclei”: HDAB-DAB [sensitivity: 40%, size: 3 µm]; (2) the setting for “Separate Nucleus Type”: H&E-hematoxylin [sensitivity: 18.3%, size: 4 µm]; (3) the setting for “Detection of Membranes”: inactive; (4) the setting for “Gene Probes”: inactive. The ratio of the number of Ki67-positive cells to the total number of cells evaluated was calculated as the Ki67 LI. Determination of invasive lesions was evaluated independently by two senior pathologists.

### Preparation of single-cell nuclei from an FFPE breast-*cancer* tissue

Three 20-µm sections were serially cut from an FFPE tissue block. The sections were deparaffinized twice in 1 ml xylene for 10 min and rehydrated by immersion in 1 ml 100% ethanol followed by 1 ml 50% ethanol and 1 ml distilled water for 3 min each. For heat-induced antigen retrieval, 1 ml of a tenfold dilution of Histo VT One (Nacalai Tesque, Inc., Kyoto, Japan) in distilled water was added to the tissues which were incubated on a heat block at 98 °C for 40 min. After incubation, the tissues were allowed to stand at room temperature for 20 min and the retrieval reagent was removed. The tissues were incubated in 0.25 ml of thrombin reagent (25 mM Tris–HCl pH 7.4, 150 mM NaCl, 1000 KU/l thrombin (FUJIFILM Wako Pure Chemical Corp.), 10 mM CaCl_2_) at 37 °C for 20 min. The enzyme-treated tissues were then crushed using a rotary homogenizer (RP-10; Sysmex Corporation, Hyogo, Japan) at 10,000 rpm for 1 min in 1 ml of TBS on ice. Suspended particles were centrifuged in 10,000 × *g* at 4 °C for 5 min, and the supernatant was removed. The pellet in 1 ml of Tris-buffered saline (TBS) was resuspended by an ultrasonic homogenizer (VCX130PB; Sonics & Materials, Inc., Newtown, CT, USA) at an amplitude of 20% for 1 min on ice and particles including single-cell nuclei were recovered as a pellet by the centrifugation in 10,000 × *g* at 4 °C for 5 min.

### Flowcytometry

Particles including single-cell nuclei were suspended in blocking buffer (25 mM Tris–HCl pH 7.4, 150 mM NaCl, 10% normal goat serum) and allowed to stand at room temperature for 30 min. Immunofluorescence staining was performed using Ki67 antibody (clone MIB-1, 1:10 dilution) and a pan-CK antibody (rabbit polyclonal antibody, 1:20 dilution; Abcam. Plc, Cambridge, UK) as the primary antibody, and anti-mouse antibody with Alexa Fluor 647 and anti-rabbit antibody Alexa Fluor 488 (each 1:500 dilution; ThermoFisher Scientific, Inc., Waltham, MA, USA). The reaction time of the primary and secondary antibodies was 37 °C for 15 min and room temperature for 15 min, respectively. For DNA staining, 5 µl of 4′,6-diamidino-2-phenylindole, dihydrochloride (DAPI) solution (FUJIFILM Wako Pure Chemical Corporation) was added to the samples after 10 min in addition to the secondary antibody. Mouse IgG1 (Agilent Technologies, Inc.) and rabbit IgG1 (Cell Signaling Technology Inc., Danvers, MA, USA) at the same concentration as each primary antibody were used as an isotype-matched control. Stained particles were washed thrice and passed through a 40 μm strainer before measurement with a flow cytometer. Flow cytometry was performed using a Novocyte Quanteon flow cytometer (Agilent Technologies, Inc.**)** or a CyFlow Space high-performance multi-laser flow cytometer (Sysmex Corporation), and analysed by FlowJo software version 10.6.2 (Becton, Dickinson and Company, Franklin Lakes, NJ, USA). The positivity of Ki67 and CK were calculated as the ratio of the number of positive cells in the evaluated total cells using the 95th percentile value of isotype-matched control as a cut-off.

### Immunofluorescence microscopy

Some of the stained cells subjected to flow cytometry analysis were observed under a BZ-X810 fluorescence microscope (Keyence Corp., Osaka, Japan). A DAPI filter (Ex 360 nm, Em 460), a GFP filter (Ex 470 nm, Em 525) and a Cy5 filter (Ex 620 nm, Em 700) were used during observation. A 20 × objective lens was used. Integrin β1 antibody (clone 4B7, 1:200 dilution; GeneTex, Inc., Irvine, CA, USA) and E-cadherin antibody (clone HECD-1, 1:100 dilution; Abcam) as the primary antibody were used to observe cells recovered from tissue.

### Statistical analysis

In Fig. 3B, to confirm the effect of thrombin treatment on Ki67 positivity, the statistical difference between groups was estimated by a paired Student’s *t* test and statistical significance was taken as *P* < 0.05. Spearman’s rank correlations were used to estimate concordance between methods. All statistical analyses were performed using MedCalc version 16.8 (MedCalc Software, Ostend, Belgium).

### Supplementary Information

Additional file 1: Figures S1-S13. Fig. S1 Bivariate FCM analysis of Ki67and DNA contentin FFPE breast cancer tissues. Fig. S2 Number of digestive enzyme cleavage sites in Ki67 isoforms, cytokeratins and collagens. Fig. S3 Flow cytometry analysis of Ki67 in formalin-fixed cell lines. Fig. S4 Flow cytometric detection of ER and PgR in formalin-fixed cell lines. Fig. S5 Optimization of mechanical tissue disruption methods used for tissue dissociation. Fig. S6 State of cells by treatment time of ultrasonic homogenization. Fig. S7 Recovery of cell nuclei from a FFPE tissue by using ultrasonic homogenization only. Fig. S8 Fluorescence microscopic observation of HER2 in cells recovered from a FFPE tissue. Fig. S9 Effect for Ki67 stainability by enzymatic treatments. Fig. S10 Negative effect of enzymatic treatment on the nucleoskeleton. Fig. S11 Gating scheme calculating Ki67 positivity in AreaX. Fig. S12 FFPE blocks of normal and breast cancer tissues from the same patient. Fig. S13 FCM of Ki67 in formalin-fixed cell lines treated with type IV collagenase.

Additional file 2: Tables S1-S3. Table S1 Reproducibility of Ki67 positivity in serial sections analysed by the FCM method. Table S2 Number of cell nuclei in each gate. Table S3 Clinicopathological data of patients.

## Data Availability

All data are contained within the manuscript, including Supplemental Information.

## Abbreviations

AI: Artificial intelligence
BIG-NABCG: The International Ki67 in Breast Cancer Working Group of the Breast International Group, and the North American Breast Cancer Group
CI: Confidence interval
CK: Cytokeratin
DAPI: 4′,6-Diamidino-2-phenylindole, dihydrochloride
ER: Oestrogen receptor
FCM: Flow cytometry
FFPE: Formalin-fixed paraffin-embedded
FSC: Forward scatter
H&E: Haematoxylin and eosin
HER2: Human epidermal growth factor receptor 2
ICC: Intraclass correlation coefficient
IHC: Immunohistochemistry
LI: Labelling index
PBS: Phosphate-buffered saline
PgR: Progesterone receptor
SSC: Side scatter
TBS: Tris-buffered saline
TIL: Tumour-infiltrated lymphocytes
