# Supplementary material for: Flow cytometric analysis for Ki67 assessment in formalin-fixed paraffin-embedded breast cancer tissue
Source: BMC Biol. 2024 Aug 26;22:181. doi: 10.1186/s12915-024-01980-4 (PMC11346000; doi:10.1186/s12915-024-01980-4)
Supplement: Supplementary file 1 — Additional file 1: Figures S1-S13. Fig. S1 Bivariate FCM analysis of Ki67and DNA contentin FFPE breast cancer tissues. Fig. S2 Number of digestive enzyme cleavage sites in Ki67 isoforms, cytokeratins and collagens. Fig. S3 Flow cytometry analysis of Ki67 in formalin-fixed cell lines. Fig. S4 Flow cytometric detection of ER and PgR in formalin-fixed cell lines. Fig. S5 Optimization of mechanical tissue disruption methods used for tissue dissociation. Fig. S6 State of cells by treatment time of ultrasonic homogenization. Fig. S7 Recovery of cell nuclei from a FFPE tissue by using ultrasonic homogenization only. Fig. S8 Fluorescence microscopic observation of HER2 in cells recovered from a FFPE tissue. Fig. S9 Effect for Ki67 stainability by enzymatic treatments. Fig. S10 Negative effect of enzymatic treatment on the nucleoskeleton. Fig. S11 Gating scheme calculating Ki67 positivity in AreaX. Fig. S12 FFPE blocks of normal and breast cancer tissues from the same patient. Fig. S13 FCM of Ki67 in formalin-fixed cell lines treated with type IV collagenase. [file 12915_2024_1980_MOESM1_ESM.docx]

**Supplementary figures**


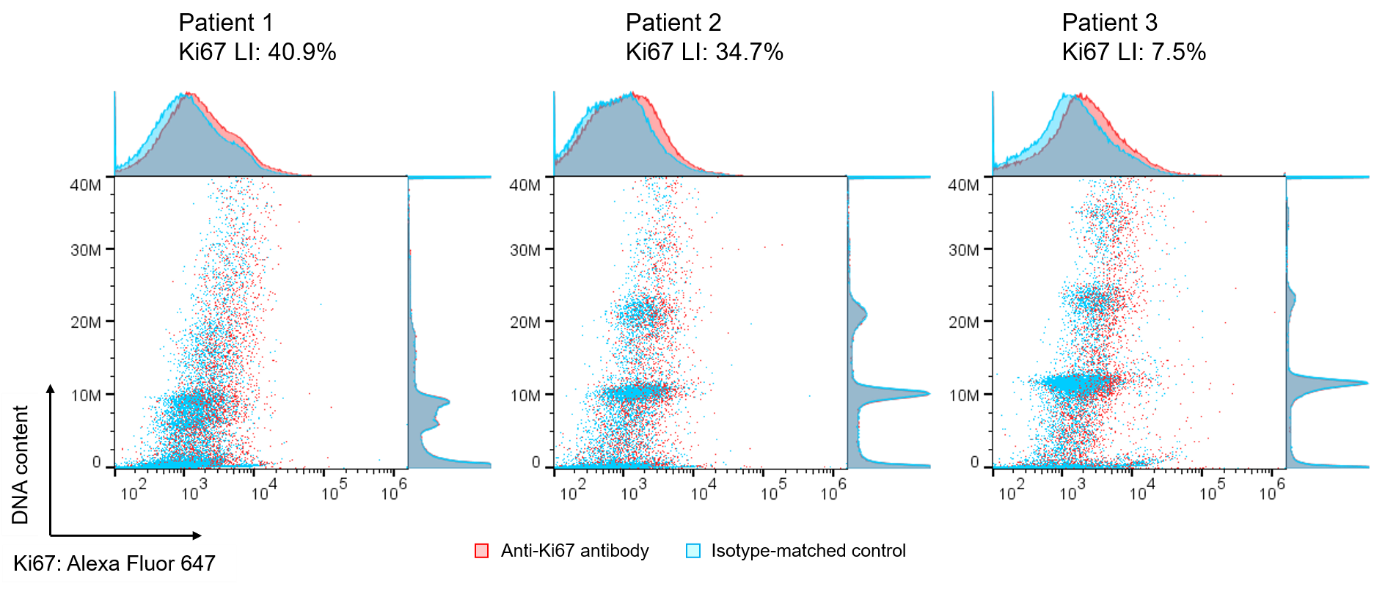


**Fig. S1** Bivariate FCM analysis of Ki67 (X-axis) and DNA content (Y-axis) in FFPE breast cancer tissues. Cells were recovered from FFPE tissues treated with 0.25 ml of trypsin reagent (25 mM Tris-HCl pH 7.4, 150 mM NaCl, 2.5 mg/ml trypsin (Worthington Biochemical Corporation, Lakewood, NJ, USA)) at 37°C for 20 min after various pretreatment methods. Tissues were then stained with DAPI and MIB-1 antibody. No gating was performed in data analysis. Red dots and curves show cells incubated with MIB-1 antibody. Blue dots and curves correspond to the background level of cells treated with isotype-matched control.


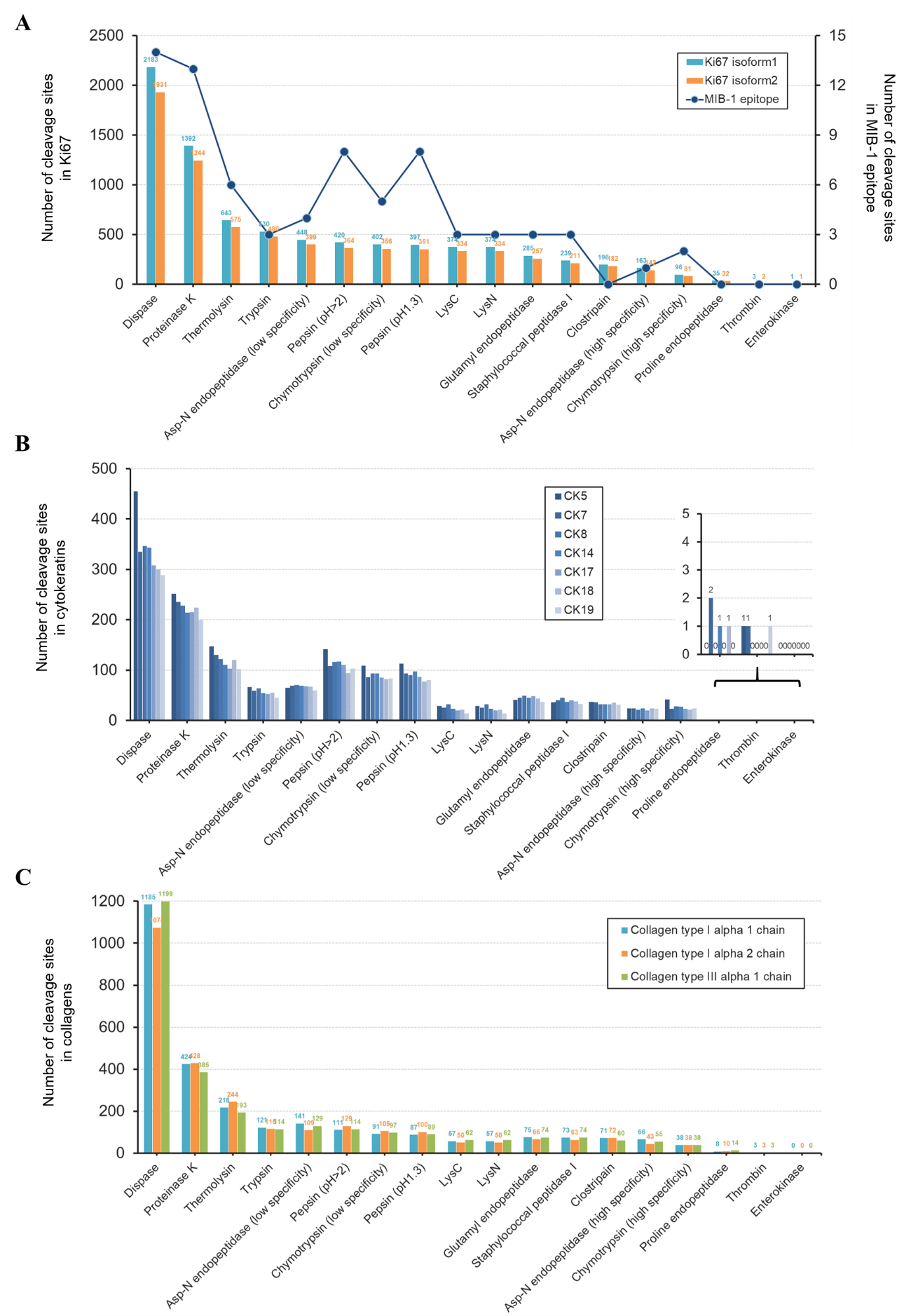


**Fig. S2** Number of digestive enzyme cleavage sites. The amino acid sequences of Ki67 isoform 1, isoform 2 (**A**), cytokeratin 5, 7, 8, 14, 17, 18, 19 (**B**), collagen type I α1 chain, α2 chain and type III α1 chain (**C**) were obtained from UniProt (https://www.uniprot.org/). Except for dispase, the number of cleavage sites for each protein was calculated using peptide cutter (https://web.expasy.org/peptide_cutter/).


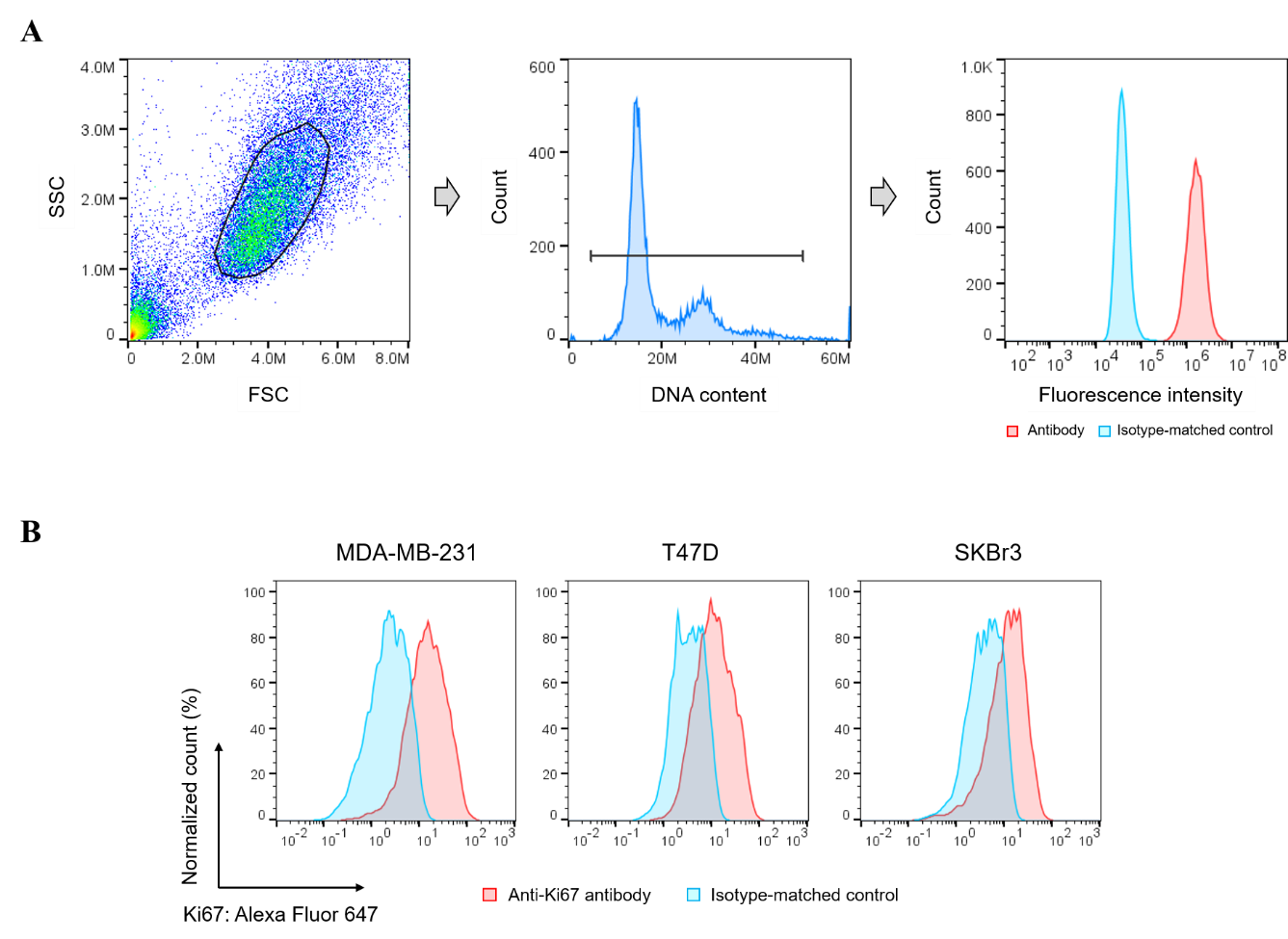


**Fig. S3** Flow cytometry (FCM) analysis of Ki67 in formalin-fixed cell lines. **A** Gating scheme. The major populations were gated on the FSC-SSC scatterplots, and debris was eliminated on the DNA content chart. Then, a composite chart was created from the antibody and isotype-matched control. **B** Detection of Ki67 in cell lines treated with proline endopeptidase. Formalin-fixed cells after an antigen retrieval were incubated in 0.25 ml of proline endopeptidase reagent (25 mM Tris-HCl pH 7.4, 150 mM NaCl, 2.0 mg/ml proline endopeptidase (TOYOBO Co., Ltd., Osaka, Japan)) at 37°C for 20 min. Cells were stained by DAPI and MIB-1 antibody. FCM analysis was performed by using CyFlow space. Red curves showed cells incubated with MIB-1 antibody. Blue curves correspond to the background level of cells treated with isotype-matched control.


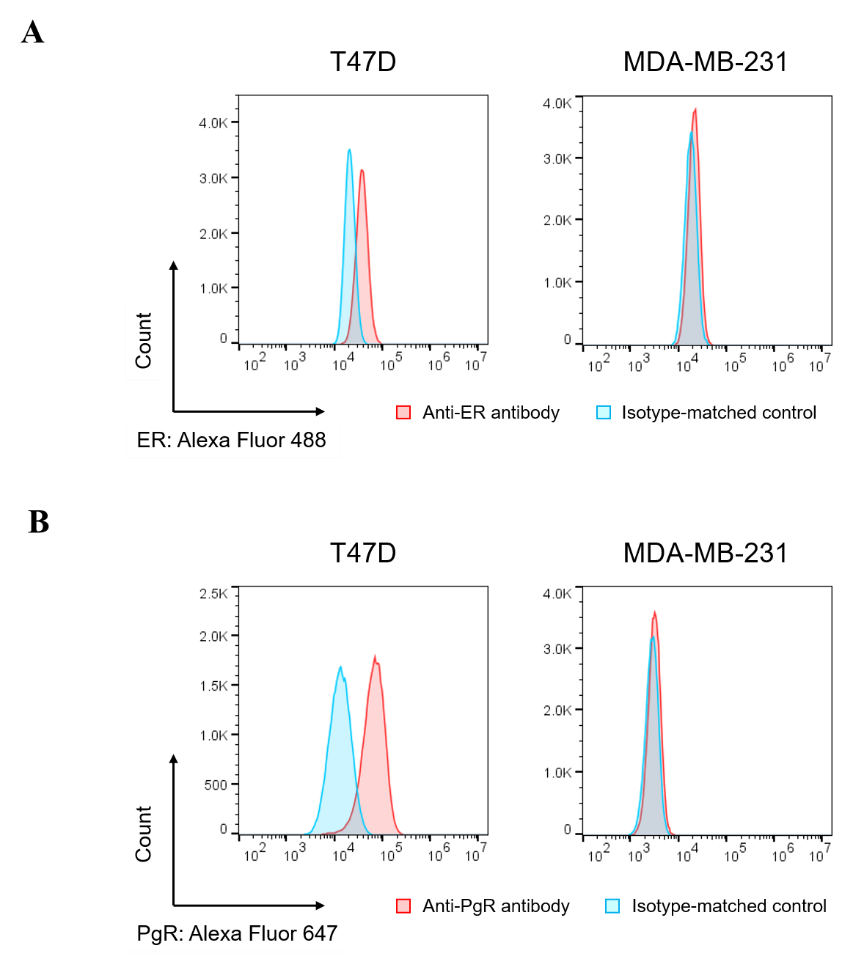


**Fig. S4** Flow cytometric detection of ER and PgR in formalin-fixed cell lines. Formalin-fixed cells after antigen retrieval were incubated in TBS buffer containing thrombin. Reactants were double-stained with ER (**A**, clone EP1, 1:10 dilution; Agilent) and PgR (**B**, clone PgR1294, 1:20 dilution; Agilent) antibodies. Mouse IgG1 and rabbit IgG1 at same concentration were used as an isotype-matched control. The gating scheme to eliminate debris is shown in Additional file 1: Fig. S3A. Red curves show cells incubated with ER or PgR antibody. Blue curves correspond to the background level of cells reacted with isotype-matched control.


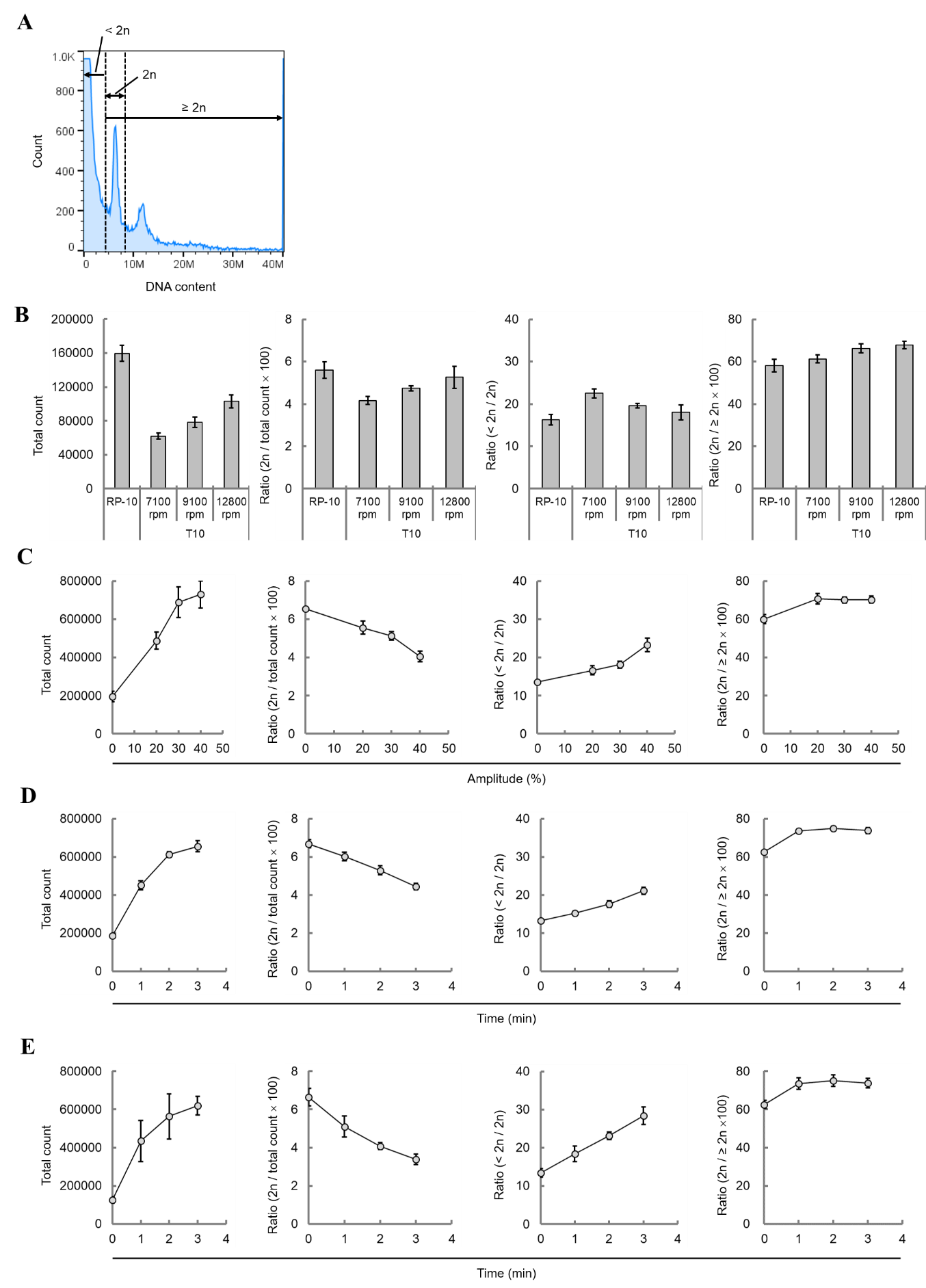


**Fig. S5** Optimization of mechanical tissue disruption methods used for tissue dissociation. **A** FFPE tissues were mechanically disrupted after deparaffinization and hydration, and the collected particles were stained with DAPI. Three gates of < 2n, 2n and ≥ 2n were created on the DNA content chart, and the number of particles in each gate were counted. **B** Two types of rotary homogenizers (RP-10: 10,000 rpm fixed, T10 basic ULTRA-TURRAX® (IKA Instruments, Staufen, Germany): three rotation speeds) were used in this experiment. The total count of recovered particles was plotted for different rotation speeds of the rotary homogenizer as the mean ± SEM (n=3) (left). Parameters of 2n/total count × 100 (middle left), < 2n/2n (middle right) and 2n/≥ 2n × 100 (right) were calculated from the population and plotted as the mean ± SEM (n=3). **C** Effect of ultrasonic homogenization for a minute after homogenization by the RP-10 rotary homogenizer. The total count of the recovered particles is plotted for different amplitudes of ultrasonic homogenizer as the mean ± SEM (n=3) (left). The other three graphs show each parameter. **D, E** Time dependency of total particle count and three parameters with 20% (**D**) and 30% (**E**) of amplitude as the mean ± SEM (n=3).

**
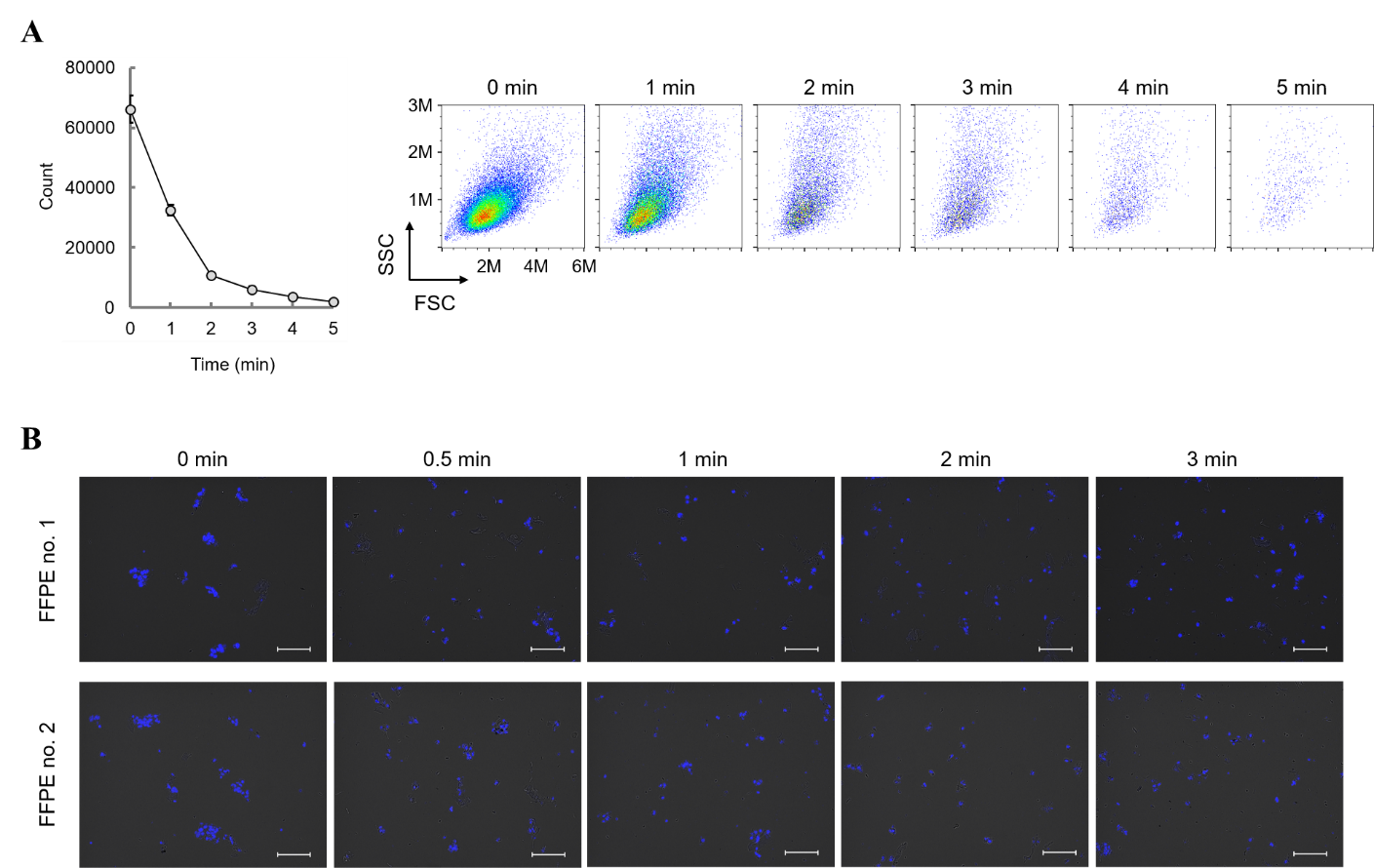
**

**Fig. S6** State of cells by treatment time of ultrasonic homogenization. **A** Formalin-fixed T47D cells were stained by DAPI after sonication at an amplitude of 20%. The number of cells per processing time was counted after elimination of debris on the DNA content chart, and their populations are also displayed on the FSC-SSC scatterplots. **B** Bright-field and fluorescence merged microscope images of DAPI-stained particle. FFPE sections obtained from two patients were sonicated at an amplitude of 20% for various times after rotary homogenization. Scale bars are 100 µm.


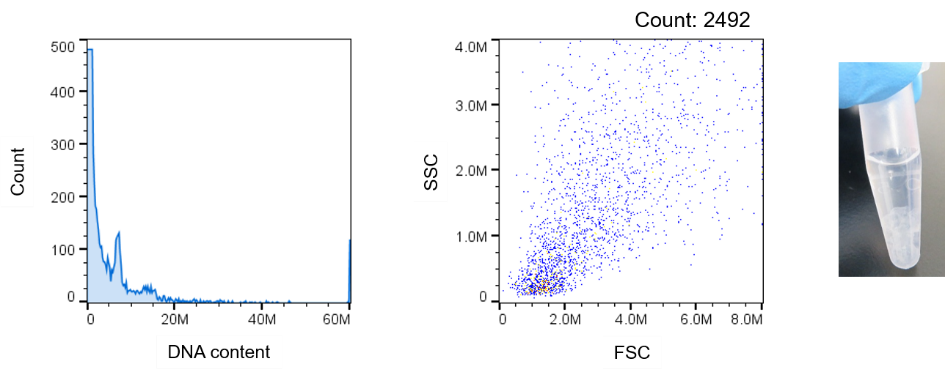


**Fig. S7** Recovery of cell nuclei from a FFPE tissue by using ultrasonic homogenization only. Sonication was only performed for 60 s after subjecting FFPE sections to various treatments. Most sections are not dissociated and remain in the tube.


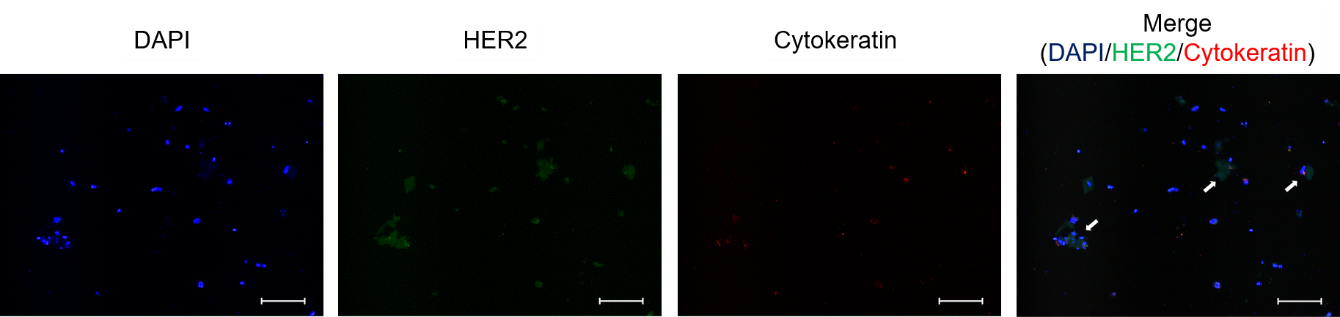


**Fig. S8** Fluorescence microscopic observation of HER2 in cells recovered from a FFPE tissue. Sections diagnosed to be HER2 positive (3+) by IHC were used in this experiment. Cells obtained by pretreatment were stained for nuclei (DAPI; blue), HER2 (polyclonal, Alexa Fluor 488; green) and cytokeratin (AE1/AE3, Alexa Fluor 647; red). Scale bars are 100 µm. Images show representative cells. White arrows show aggregates of tissue debris.


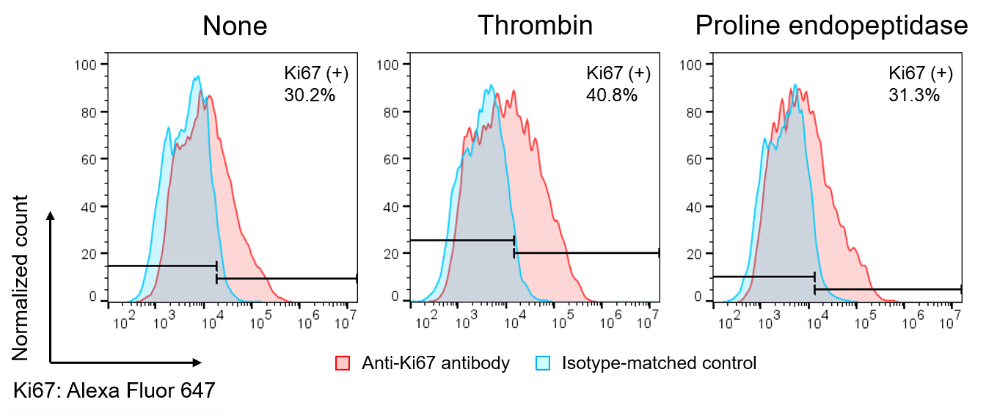


**Fig. S9** Effect for Ki67 stainability by enzymatic treatments. Representative charts of Ki67-stained cell nuclei without thrombin, with thrombin or with proline endopeptidase treatment from serially sectioned FFPE breast cancer tissues. Red curves show cell nuclei incubated with MIB-1 antibody. Blue curves correspond to the background level of cell nuclei reacted with isotype-matched control. Ki67 positivity was calculated as the proportion of Ki67-positive cell nuclei in total cell nuclei. The 95^th^ percentile of isotype-matched control was used to identify Ki67-negative and Ki67-positive cell nuclei.


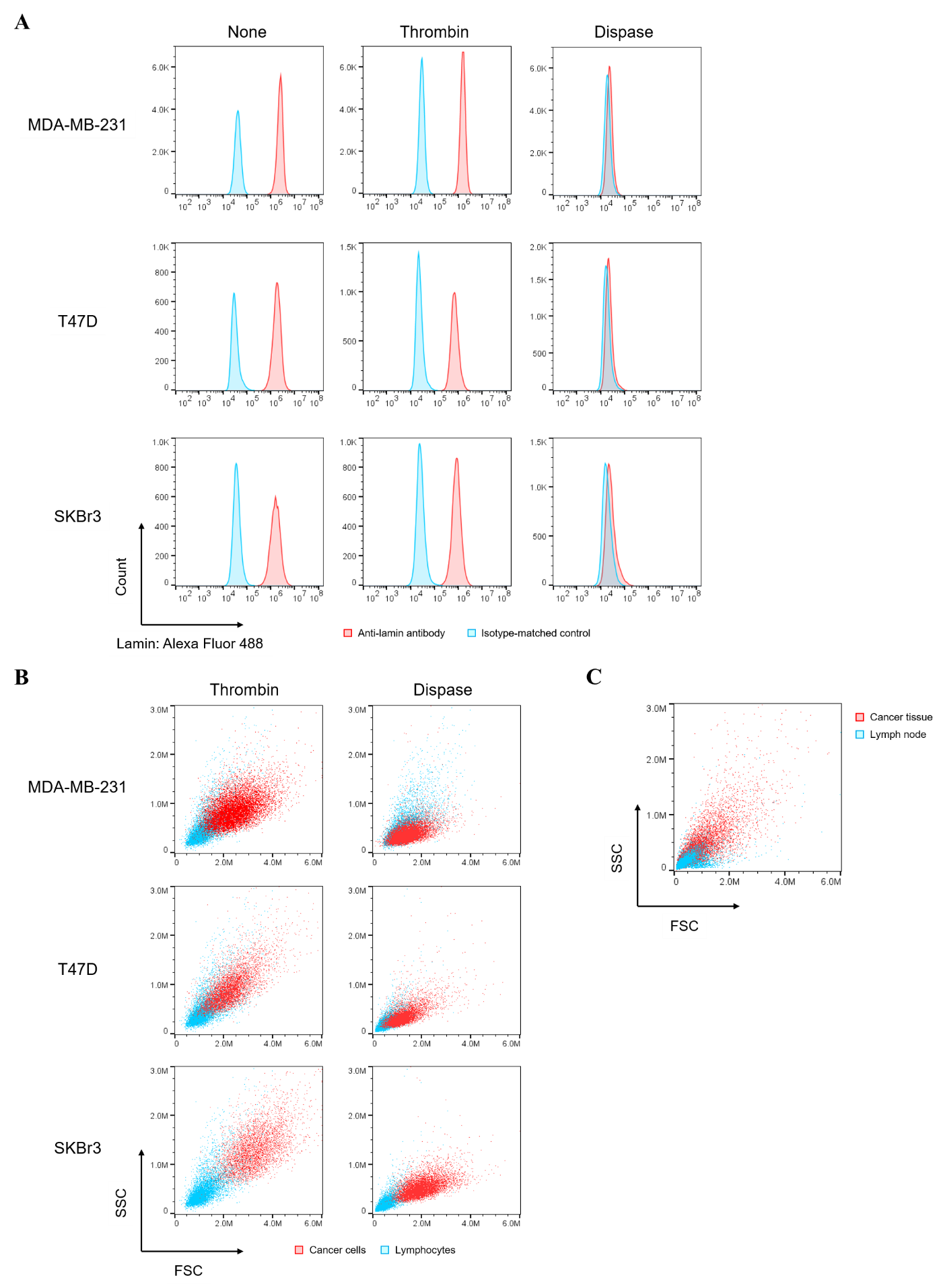


**Fig. S10** Negative effect of enzymatic treatment on the nucleoskeleton. **A** Detection of lamin in cell lines treated with digestive enzymes. After antigen retrieval, formalin-fixed cells were incubated in TBS buffer only, thrombin or dispase added TBS buffer. Reactants were stained by DAPI and lamin antibody. A gating scheme to eliminate debris is shown in Fig. S3A. Red curves show cells incubated with lamin antibody. Blue curves correspond to the background level of cells reacted with isotype-matched control. **B** Distribution of cells after enzymatic treatment on the FSC-SSC scatterplots. Red dots show formalin-fixed cancer cell lines (MDA-MB-231, T47D and SKBr3). Blue dots show formalin-fixed Jurkat cells. **C** Distribution of cell nuclei from FFPE tissues of breast cancer and lymph node treated with dispase.


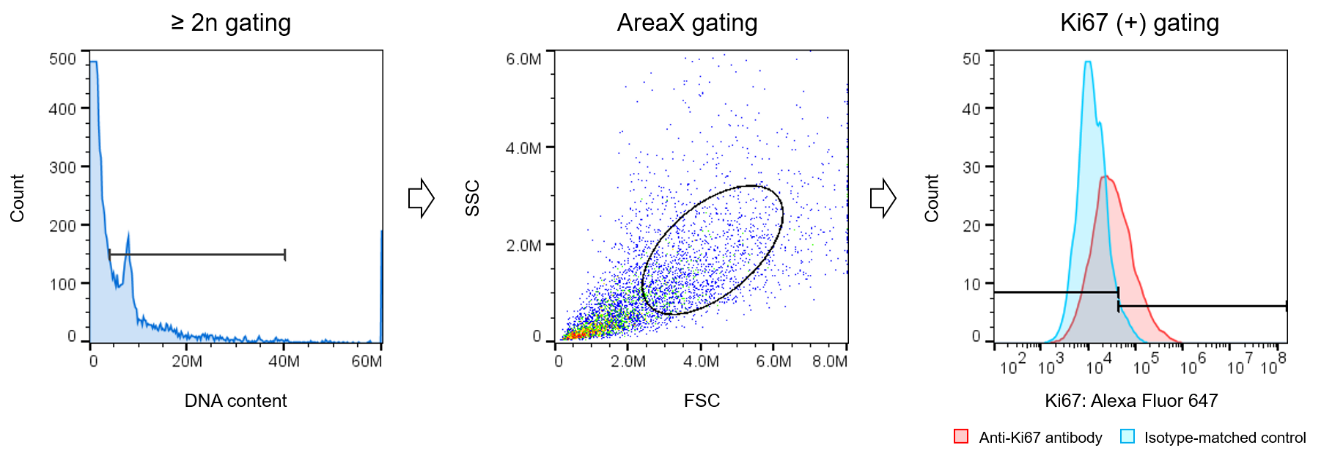


**Fig. S11** Gating scheme calculating Ki67 positivity in AreaX. The cell nuclei population was initially gated in the region with the 2n peak and more than 2n on the DNA content chart, and debris were eliminated. Then, cell nuclei within AreaX were gated to enrich cancer cells. Ki67 positivity was calculated as the ratio of Ki67 positive nuclei to the total number of cell nuclei in AreaX, using the 95^th^ of the isotype-matched control as a cut-off.


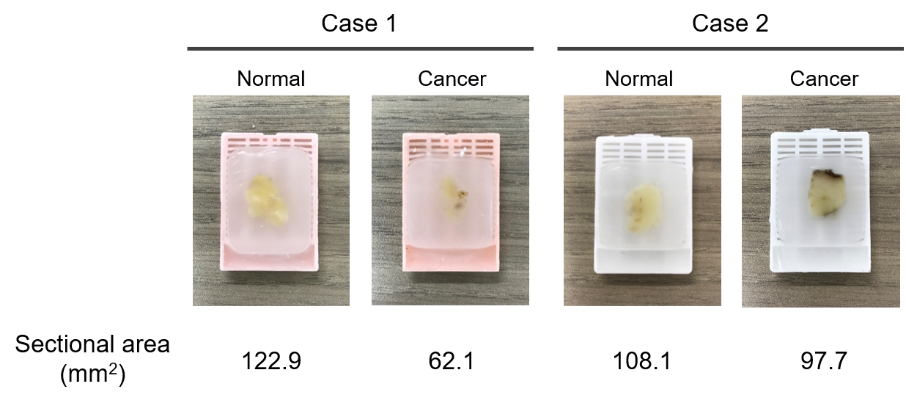


**Fig. S12** FFPE blocks of normal and breast cancer tissues from the same patient. The sectional areas were calculated by ImageJ.


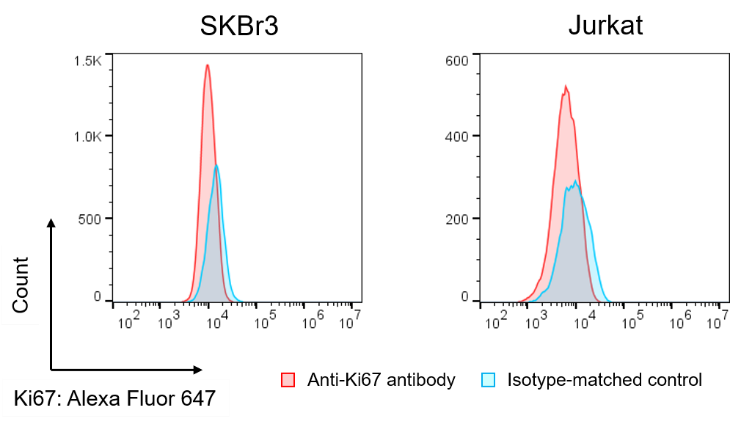


**Fig. S13** FCM of Ki67 in formalin-fixed cell lines treated with type IV collagenase. Formalin-fixed cells after antigen retrieval were incubated in 0.25 ml of type IV collagenase reagent (25 mM Tris-HCl pH 7.4, 150 mM NaCl, 2.5 mg/ml type IV collagenase (Worthington Biochemical Corporation, Lakewood, NJ, USA)) at 37°C for 20 min. Reactants were stained by DAPI and MIB-1 antibody. Red curves show cells incubated with MIB-1 antibody. Blue curves correspond to the background level of cells treated with isotype-matched control.
