# Supplementary material for: Flow cytometric analysis for Ki67 assessment in formalin-fixed paraffin-embedded breast cancer tissue
Source: BMC Biol. 2024 Aug 26;22:181. doi: 10.1186/s12915-024-01980-4 (PMC11346000; doi:10.1186/s12915-024-01980-4)
Supplement: Supplementary file 2 — Additional file 2: Tables S1-S3. Table S1 Reproducibility of Ki67 positivity in serial sections analysed by the FCM method. Table S2 Number of cell nuclei in each gate. Table S3 Clinicopathological data of patients. [file 12915_2024_1980_MOESM2_ESM.docx]

**Supplementary tables**

**Table S1.** Reproducibility of Ki67 positivity in serial sections analysed by the FCM method

|  | Ki67 positivity | | |
| --- | --- | --- | --- |
|  | Low | Middle | High |
| n | 5 | 10 | 5 |
| Mean | 8.1 | 19.2 | 42.5 |
| Max | 9.6 | 22.3 | 45.2 |
| Min | 6.5 | 17.1 | 40.7 |
| Range | 3.1 | 5.1 | 4.5 |
| SD | 0.99 | 1.76 | 1.62 |
| CV (%) | 12.2 | 9.2 | 3.8 |

SD: standard deviation; CV: coefficient of variation

**Table S2.** Number of cell nuclei in each gate

|  | AreaX | Cytokeratin | DAPI 2N only |
| --- | --- | --- | --- |
| n | 30 | 30 | 30 |
| Mean | 1885 | 1616 | 7356 |
| Max | 6342 | 5154 | 26045 |
| Min | 333 | 98 | 1137 |

**Table S3.** Clinicopathological data of patients

|  | Number of patients (%) |
| --- | --- |
| Total | 30 |
|  |  |
| Age at diagnosis (years) |  |
| Mean ± SD | 62.5 ± 13.4 |
| Range | 36 - 88 |
| Histology |  |
| Ductal carcinoma in situ | 3 (10.0) |
| Invasive ductal carcinoma | 22 (73.4) |
| Invasive lobular carcinoma | 3 (10.0) |
| Invasive micropapillary carcinoma | 1 (3.3) |
| Mucinous carcinoma | 1 (3.3) |
| Histological grade |  |
| 1 | 18 (60.0) |
| 2 | 3 (10.0) |
| 3 | 6 (20.0) |
| None | 3 (10.0) |
| Tumour size (TNM) |  |
| Tis | 3 (10.0) |
| T1 | 20 (66.7) |
| T2 | 6 (20.0) |
| T3 | 1 (3.3) |
| Lymph node metastasis |  |
| Negative | 24 (80.0) |
| Positive | 6 (20.0) |
| Ki67 (%) |  |
| < 20 | 20 (66.7) |
| ≥ 20 | 10 (33.3) |

SD: standard deviation
